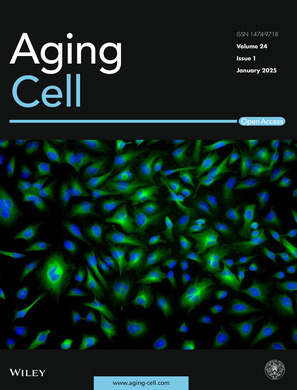# Additional Cover

**DOI:** 10.1111/acel.14480

**Published:** 2025-01-08

**Authors:** Nathalie Launay, Maria Espinosa‐Alcantud, Edgard Verdura, Gorka Fernández‐Eulate, Jon Ondaro, Pablo Iruzubieta, Maria Marsal, Agatha Schlüter, Montserrat Ruiz, Stephane Fourcade, Agustí Rodríguez‐Palmero, Miren Zulaica, Andone Sistiaga, Garazi Labayru, Pablo Loza‐Alvarez, Alejandro Vaquero, Adolfo Lopez de Munain, Aurora Pujol

## Abstract

Cover legend: The cover image is based on the article *Altered tubulin detyrosination due to SVBP malfunction induces cytokinesis failure and senescence, underlying a complex hereditary spastic paraplegia* by Aurora Pujol et al., https://doi.org/10.1111/acel.14355.